# Serum levels of VCAM‐1 are associated with survival in patients treated with nivolumab for NSCLC

**DOI:** 10.1111/eci.13668

**Published:** 2021-08-22

**Authors:** Federico Carbone, Stefano Ministrini, Aldo Bonaventura, Alessandra Vecchié, Silvia Minetti, Nicholas Bardi, Edoardo Elia, Anna Maria Ansaldo, Daniele Ferrara, Erika Rijavec, Maria Giovanna Dal Bello, Federico Biello, Giovanni Rossi, Marco Tagliamento, Angela Alama, Simona Coco, Paolo Spallarossa, Francesco Grossi, Carlo Genova, Fabrizio Montecucco

**Affiliations:** ^1^ First Clinic of internal Medicine Department of Internal Medicine University of Genoa Genoa Italy; ^2^ IRCCS Ospedale Policlinico San Martino Genoa – Italian Cardiovascular Network Genoa Italy; ^3^ Center for Molecular Cardiology Universität Zürich Schlieren Switzerland; ^4^ Internal Medicine, Angiology and Atherosclerosis Department of Medicine and Surgery Università degli Studi di Perugia Perugia Italy; ^5^ Division of Cardiology Department of Internal Medicine Pauley Heart Center Virginia Commonwealth University Richmond VA USA; ^6^ Division of Cardiology Department of Internal Medicine Città della Salute e della Scienza Turin Italy; ^7^ Medical Oncology Unit Fondazione IRCCS Ca'Granda Ospedale Maggiore Policlinico Milan Italy; ^8^ UOS Tumori Polmonari IRCCS Ospedale Policlinico San Martino Genoa Genoa Italy; ^9^ Department of Internal Medicine and Medical Specialties (DiMI) University of Genova Genoa Italy; ^10^ Cardiovascular Disease Unit IRCCS Ospedale Policlinico San Martino Genoa Genoa Italy

**Keywords:** intracellular adhesion molecule‐1, nivolumab, non‐small cell lung cancer, programmed cell‐death protein‐1, vascular cell adhesion molecule‐1

## Abstract

**Background:**

High circulating levels of cellular adhesion molecules (CAMs) in non‐small cell lung cancer (NSCLC) have been supposed to act as a negative prognostic factor. Here, we explored the predictive role of pre‐treatment levels of CAMs in previously treated patients receiving nivolumab for NSCLC.

**Materials and methods:**

Seventy one patients with advanced NSCLC, treated with nivolumab at the dose of 3 mg/kg every 14 days, were enrolled. Maximum follow‐up time was 3 years. Serum levels of Vascular Cell Adhesion Molecule‐1 (VCAM‐1) and Intracellular Adhesion Molecule‐1 (ICAM‐1) were measured at baseline and before each nivolumab administration. Endpoints of the study were a composite outcome of survival ≥2 years or absence of disease progression at the end of the follow‐up, and the overall survival.

**Results:**

Composite outcome and overall survival were positively associated with VCAM‐1 baseline levels and with the reduction of VCAM‐1 during the treatment. After adjustment for potential confounders, the change in VCAM‐1 serum levels during the treatment was an independent predictor of overall survival.

**Conclusions:**

High baseline serum levels of VCAM‐1 are associated with a longer survival in patients treated with nivolumab as second line treatment for NSCLC. Surviving patients experience also a significant reduction in CAMs expression during the treatment. Hence, CAMs might be promising prognostic factors in patients with NSCLC underoing immunotherapy.

## 1 INTRODUCTION

As a pivotal process in neo‐angiogenesis and metastatic dissemination of many different malignancies, the interaction between integrins and cellular adhesion molecules (CAMs) is constituting a promising therapeutic target. In vitro studies, indeed, reported that vascular cellular adhesion molecule‐1 (VCAM‐1) is overexpressed in non‐small cell lung cancer (NSCLC) cells and its expression is associated with invasive phenotype^1^; moreover, low plasma levels of intracellular adhesion molecule‐1 (ICAM‐1) seem to be associated with a better response to conventional chemotherapy.^2^

No previous study has explored whether interaction exists between CAMs expression and treatment with immune checkpoint inhibitors (ICIs). This class of drugs has substantially improved the prognosis of patients with NSCLC and is currently recommended also as first‐line treatment, alone or in combination with chemotherapy, for advanced disease.^3^

Several authors hypothesized that an appropriate stratification of patients, according to their likelihood of response, could improve the outcome of immunotherapy for patients with NSCLC. In this regard, serum inflammatory biomarkers are a promising field of research because they are little expensive, easily performed and can be combined with other predictors in a probability score.^4^

With this sub‐analysis of a previously published, single‐arm, open labelled cohort,^5^ we tested the prognostic value of the pre‐treatment levels of VCAM‐1 and ICAM‐1 in patients receiving nivolumab and their clinical relevance in terms of a composite outcome, defined as a survival ≥2 years or absence of disease progression at the closing of follow‐up, and overall survival (OS) during immune‐therapy. Furthermore, we investigated the potential change of serum levels of CAMs during nivolumab treatment.

## 2 MATERIALS AND METHODS

### 2.1 Patients

Original cohort was composed of 74 patients (age ≥18 years) with advanced NSCLC (stage IIIb or IV at diagnosis, according to the Tumor‐Nodes‐Metastases classification v.7.0) and a history of disease persistence or progression after at least one line of conventional treatment, consecutively referred to the Lung Cancer Unit of IRCCS Ospedale Policlinico San Martino in Genoa (Italy), between 1 April 2015 and 30 June 2016. Inclusion and exclusion criteria were previously described.^5^, ^6^ Nivolumab was provided by Bristol‐Myers‐Squibb within the Italian expanded access programme in NSCLC and was administered at the dose of 3 mg/kg every 14 days. Nivolumab was administered until onset of unacceptable toxicity, patient refusal, radiologically confirmed disease progression, or upon reaching 96 weeks from the start of treatment. Biochemical data were collected for the first 5 cycles of treatment (total 70 days). Follow‐up was closed after 3 years (1095 days) from the enrolment of the first patient. Blood samples were collected before each administration of nivolumab, immediately centrifuged to separate sera and then stored at −80°C. Pre‐treatment sera were available for 71 out of 74 patients. The process of data collection is summarized in Figure S1. All patients gave written informed consent before entering the study. The protocol was approved by the local Ethics Committee of ‘IRCCS Ospedale Policlinico San Martino’. All performed procedures were in accordance with the Declaration of Helsinki of 1964 and its later amendments for studies involving humans.

### 2.2 Measurements

Serum levels of VCAM‐1 and ICAM‐1 were measured by enzyme‐linked immunosorbent sandwich assay (ELISA) with double‐automated colorimetric reading, following manufacturer's instructions (R&D Systems). The low detection range was 15.625 pg/ml for VCAM‐1 and 31.25 pg/ml for ICAM‐1. Intra‐ and inter‐assay coefficient of variation was below 8% for both markers.^7^, ^8^ Blood cell count and biochemical analysis of sera were performed with routine automated diagnostic techniques (LH750 and DxC700 CoreLab, Beckman Coulter Italia).

Performance status was evaluated according to the Eastern Cooperative Oncology Group scale of Performance Status (ECOG‐PS).^9^ Early response to treatment was instead evaluated by computed tomography scan after the 4^th^ cycle of treatment (8 weeks), according to the Response Evaluation Criteria in Solid Tumors (RECIST) version 1.1.^10^ A score was attributed to each category: 0=death before response assessment; 1 = progression of disease (PD); 2 = stable disease (SD); 3 = partial response (PR); and 4 = complete response (CR).

### 2.3 Endpoint adjudication and power study calculation

The primary endpoint was to assess the correlation between the circulating values of VCAM‐1 and ICAM‐1, and a composite outcome, defined as a survival ≥2 years or absence of disease progression at the closing of follow‐up. The secondary endpoint was to assess the correlation between circulating CAMs and OS.

Based on existing literature,^2^ a survival rate difference of 0.4 was expected between patients with high and low levels of ICAM‐1 (cut‐off 260.5 ng/ml) after 18 months of follow‐up. To reach a desired power of 0.8, a sample size of 136 patients was required. Therefore, this study could be underpowered and should be regarded as a pilot study.

### 2.4 Statistical analysis

Statistical analysis was performed using IBM spss statistic software package, version 23.0 (IBM Corp.). Values of quantitative variables are expressed as mean (standard deviation) for normally distributed variables and as median (interquartile range) for non‐normally distributed variables. Normality of distributions was assessed using the Kolmogorov‐Smirnov test. Categorical variables were expressed as number (%). Variations were calculated as (final value–initial value). Statistical significance of variations was evaluated using a paired *t*‐test for normally distributed variables and the Wilcoxon test for non‐normally distributed variables. Bonferroni correction was employed for repeated measures. Bivariate correlations coefficients were calculated using the Pearson and Spearman correlation tests, for normally and non‐normally distributed variables, respectively. Potential confounders were selected through univariate regression models. Only parameters reaching the statistical significance (*p* < .05) were introduced in multivariate models. Multivariate logistic regression and Cox regression models were used to test independent predictors. Results were expressed with 95% confidence as odds ratio (OR) and hazard ratio (HR), respectively. Variables were log‐transformed, when necessary. Goodness‐of‐fit of the logistic regression models was tested through a receiving operator curve (ROC).

## 3 RESULTS

### 3.1 Baseline characteristics

Clinical, haematological and biochemical characteristics of patients at enrolment are reported in Tables S1 and S2. Pre‐treatment levels of VCAM‐1 were significantly higher in subjects who achieved the composite outcome and in subjects who were still alive at the closing of the follow‐up, whereas no significant difference was detected for baseline levels of ICAM‐1. Pre‐treatment levels of both VCAM‐1 and ICAM‐1 were higher in patients with adenocarcinoma than in patients with squamous cells carcinoma. Circulating ICAM‐1 was positively associated with the expression of PD‐L1 (Table 1). Baseline characteristics of the cohort, according to the study endpoints, are reported in Table 2.

**TABLE 1:** Comparisons of ICAM‐1/VCAM‐1 values at the enrolment, according to different clinical outcomes and potential confounders

	ICAM−1		VCAM−1	
	ng/ml (IQR)	*p*‐value	ng/ml (IQR)	*p*‐value
Sex (*n* = 71)				
Male	195 (152–263)	.055	308 (158–505)	.687
Female	250 (186–410)		340 (168–522)	
Histology^a^ (*n* = 68)				
ADC	223 (171–323)	.**041**	362 (197–507)	.**009**
SCC	176 (126–205)		175 (76–271)	
Number of metastatic sites (*n* = 70)				
1	232 (180–253)	.351	394 (252–577)	.741
2	242 (136–343)		255 (183–597)	
3	200 (154–254)		338 (151–500)	
4	185 (155–263)		222 (86–481)	
5	221 (141–638)		292 (182–386)	
6 or more	355 (194–621)		439 (133–728)	
Liver metastases (*n* = 70)				
No	210 (149–275)	.172	270 (171–501)	.879
Yes	211 (186–441)		347 (122–515)	
PD‐L1 expression (*n* = 33)				
<1%	173 (122–214)	.**003**	246 (190–368)	.065
1%–10%	329 (219–402)		507 (309–564)	
10%–49%	437 (264–600)		420 (260–563)	
≥50%	354 (‐)		628 (‐)	
ECOG PS (*n* = 71)				
0	252 (193–363)	.135	369 (170–508)	.666
1	198 (140–277)		278 (165–491)	
2	186 (99–470)		200 (177–667)	
3	149 (–)		143 (–)	
Prior lines of treatment (*n* = 67)				
1	210 (176–280)	.663	363 (163–493)	.816
2	172 (148–363)		354 (170–582)	
3	241 (156–450)		292 (173–608)	
4	239 (159–272)		182 (134–414)	
5	–		–	
6	151 (109–151)		151 (109–151)	
Smoking (*n* = 65)				
Never	225 (105–472)	.953	225 (196–621)	.757
Former	213 (171–268)		368 (169–540)	
Active	210 (155–318)		278 (161–496)	
RECIST First response (*n* = 67)				
Early death	196 (152–304)	.180	357 (178–515)	.217
PD	186 (132–275)		225 (120–412)	
SD	230 (172–329)		408 (206–596)	
PR	262 (243–419)		465 (305–505)	
CR	–		–	
Composite outcome (*n* = 71)				
No	205 (271–157)	.304	270 (132–473)	.**023**
Yes	252 (164–363)		491 (200–643)	
Death (*n* = 71)				
No	508 (364–823)	.143	305 (199–380)	.**007**
Yes	270 (154–461)		197 (160–273)	

Continuous variables are presented as median with interquartile range (IQR). Analyses were drawn by Mann‐Whitney and Kruskal‐Wallis tests, as appropriate. Statistically significant p‐values are highlighted in bold.

Abbreviations: ADC, adenocarcinoma; CR: complete response; ECOG PS, Eastern Cooperative Oncology Group Performance Status; PD, progression disease; PD‐L1, programmed death‐ligand 1; PR, partial response; RECIST, response evaluation criteria in solid tumours; SCC, squamous cell carcinoma; SD, stable disease.

^a^Patients with other histological types of carcinoma were excluded (*n* = 3).

**TABLE 2:** Clinical parameters of the cohort according to the endpoints

	Composite outcome^a^			Survival		
	Yes (*n* = 14)	No (*n* = 57)	*p‐value*	Yes (*n* = 9)	No (*n* = 62)	*p*‐value
Age, years [IQR]	67.5 [62.0–76.0]	70.0 [62.0–75.8]	.628	66.0 [60.0–72.0]	70.0 [62.0–76.0]	.284
Sex, male (%)	10 (71.4)	40 (70.2)	.603	7 (77.8)	43 (69.4)	.716
Histology*						
ADC, *n* (%)	11 (84.6)	41 (74.5)	.718	7 (87.5)	45 (75.0)	.670
SCC, *n* (%)	2 (15.4)	14 (25.5)		1 (12.5)	15 (25.0)	
ECOG PS						
0, *n* (%)	8 (57.1)	15 (26.3)	.055	5 (55.6)	18 (29.0)	.055
1, *n* (%)	4 (26.7)	38 (67.9)		3 (33.3)	39 (62.9)	
2, *n* (%)	2 (13.3)	3 (5.4)		1 (11.1)	4 (6.5)	
3, *n* (%)	‐	1 (1.8)		‐	1 (1.6)	
Number of metastatic sites						
1	3 (23.1)	5 (8.8)	.**022**	2 (22.2)	6 (9.8)	.488
2	6 (46.2)	10 (17.5)		4 (44.4)	12 (19.7)	
3	2 (15.4)	19 (33.3)		2 (22.2)	19 (31.1)	
4	1 (7.7)	12 (29.1)		1 (11.1)	12 (19.7)	
5	‐	6 (10.5)		‐	6 (9.8)	
6 or more	1 (7.7)	5 (8.8)		‐	6 (9.8)	
Liver metastases						
No	10 (76.9)	38 (66.7)	.742	7 (77.8)	41 (67.2)	.709
Yes	3 (23.1)	19 (33.3)		2 (22.2)	20 (32.8)	
PD‐L1 expression						
<1%	3 (42.9)	19 (73.1)	.**009**	1 (25.0)	21 (72.4)	.**007**
1%–10%	4 (57.1)	2 (7.7)		3 (75.0)	3 (10.3)	
10%–49%	0 (0.0)	4 (15.4)		‐	4 (13.9)	
≥50%	0 (0.0)	1 (3.8)		‐	1 (3.4)	
Prior lines of treatment						
1, *n* (%)	6 (40.0)	26.0 (50.0)	.537	4 (44.4)	28 (48.3)	.499
2, *n* (%)	4 (28.6)	10 (20.8)		2 (22.2)	13 (22.4)	
3, *n* (%)	3 (20.0)	9 (17.3)		2 (22.2)	10 (17.2)	
4, *n* (%)	‐	6 (11.5)		‐	6 (10.3)	
5, *n* (%)	‐	‐		‐	‐	
6, *n* (%)	1 (6.7)	1 (1.9)		1 (11.1)	1 (1.7)	
Smoki*n*g						
No, *n* (%)	1 (6.7)	6 (12.0)	.537	‐	7 (12.5)	.114
Active, *n* (%)	6 (42.9)	28 (54.9)		3 (33.3)	31 (55.4)	
Previous, *n* (%)	7 (46.7)	17 (34.0)		6 (66.6)	18 (32.1)	
First response						
Early death, *n* (%)	‐	18 (31.6)	.**044**	‐	18 (29.0)	.**001**
PD, *n* (%)	4 (28.6)	23 (40.4)		‐	27 (43.5)	
SD, *n* (%)	8 (57.1)	12 (21.1)		7 (7.8)	13 (21.0)	
PR, *n* (%)	2 (14.3)	4 (7.0)		2 (22.2)	4 (6.5)	
CR, *n* (%)	‐	‐		‐	‐	

Comparisons were drawn by Mann‐Whitney U test or χ‐square test. Statistically significant p‐values are highlighted in bold.

Abbreviations: ADC, adenocarcinoma; CR, complete response; ECOG PS, Eastern Cooperative Oncology Group Performance Status; PD, progression disease; PD‐L1, programmed death‐ligand 1; PR, partial response; SCC, squamous cell carcinoma; SD, stable disease.

^a^Composite outcome is defined as an overall survival ≥2 years or absence of disease progression at the closing of follow‐up.

^b^Patients with other histological types of carcinoma were excluded (n = 3).

Baseline levels of VCAM‐1 and ICAM‐1 were significantly correlated with each other (Figure S2A). No significant correlation was instead detected between baseline levels of VCAM‐1 or ICAM‐1 and blood levels of leukocytes, haemoglobin, markers of kidney function, liver function and inflammation (Table S3). Baseline levels of ICAM‐1—but not VCAM‐1—also correlated with a better performance status at baseline (Figure S2B,C). A significant correlation was observed between the neutrophils‐to‐lymphocytes ratio (NLR) and the OS (HR: 1.024, 95% CI: 1.011–1.037, *p* < .001; Table 3).

**TABLE 3:** Comparison of potential determinants of composite outcome and overall survival (OS). Results are expressed as odds ratio (OR) and hazard ratio (HR)

		OR	95% C.I.	*p‐value*
Composite outcome				
VCAM−1 baseline (ng/ml)		1.003	1.001–1.006	.**013**
ΔVCAM−1 [0–4 weeks] (ng/ml)		0.993	0.988–0.998	.**006**
NLR		0.929	0.814–1.060	.273
PD‐L1 >1%		3.619	0.642–20.407	.145
N. of metastatic sites				.277
1	*n* = 8	Ref	Ref	‐
2	*n* = 16	1.000	0.173–5.772	.999
3	*n* = 21	0.175	0.023–1.353	.095
4	*n* = 13	0.139	0.011–1.679	.121
5	*n* = 6	0.000	‐	.999
>5	*n* = 6	0.333	0.025–4.401	.404
RECIST				.159
PD	*n* = 28	Ref	Ref	‐
SD	*n* = 20	0.348	0.047–2.576	.301
PR	*n* = 6	1.333	0.196–9.083	.769

		HR	95% C.I.	*p‐value*
Overall survival (days)				
VCAM−1 baseline (ng/ml)		0.715	0.517–0.989	.**043**
ΔVCAM−1 [0–4 weeks] (ng/ml)		1.002	1.001–1.003	.**004**
NLR		1.024	1.011–1.037	**<.001**
PD‐L1 >1%		0.504	0.218–1.165	.109
N. of metastatic sites				.**010**
1	*n* = 8	Ref	Ref	‐
2	*n* = 16	0.844	0.315–2.261	.735
3	*n* = 21	1.855	0.747–4.765	.179
4	*n* = 13	2.080	0.776–5.576	.140
5	*n* = 6	5.595	1.729–18.112	.**004**
>5	*n* = 6	1.872	0.563–5.639	.362
RECIST				.**003**
PD	*n* = 28	Ref	Ref	‐
SD	*n* = 20	2.385	0.828–6.869	.107
PR	*n* = 6	0.756	0.241–2.351	.624

The effects on probability of composite outcome were tested with binary logistic regression. The effects on overall survival were tested with the Cox proportional regression. Censoring event is: death.

HR and OR are calculated for one unit increase of continuous variables. Statistically significant p‐values are highlighted in bold.

Abbreviations: NLR, neutrophils‐to‐lymphocytes ratio; PD, progression of disease; PD‐L1, programmed death ligand‐1; PR, partial remission; RECIST, Response Evaluation Criteria in Solid Tumors; SD, stable disease; VCAM‐1, vascular cellular adhesion molecule‐1.

### 3.2 Effect of treatment with nivolumab on circulating CAMs

**FIGURE 1:**
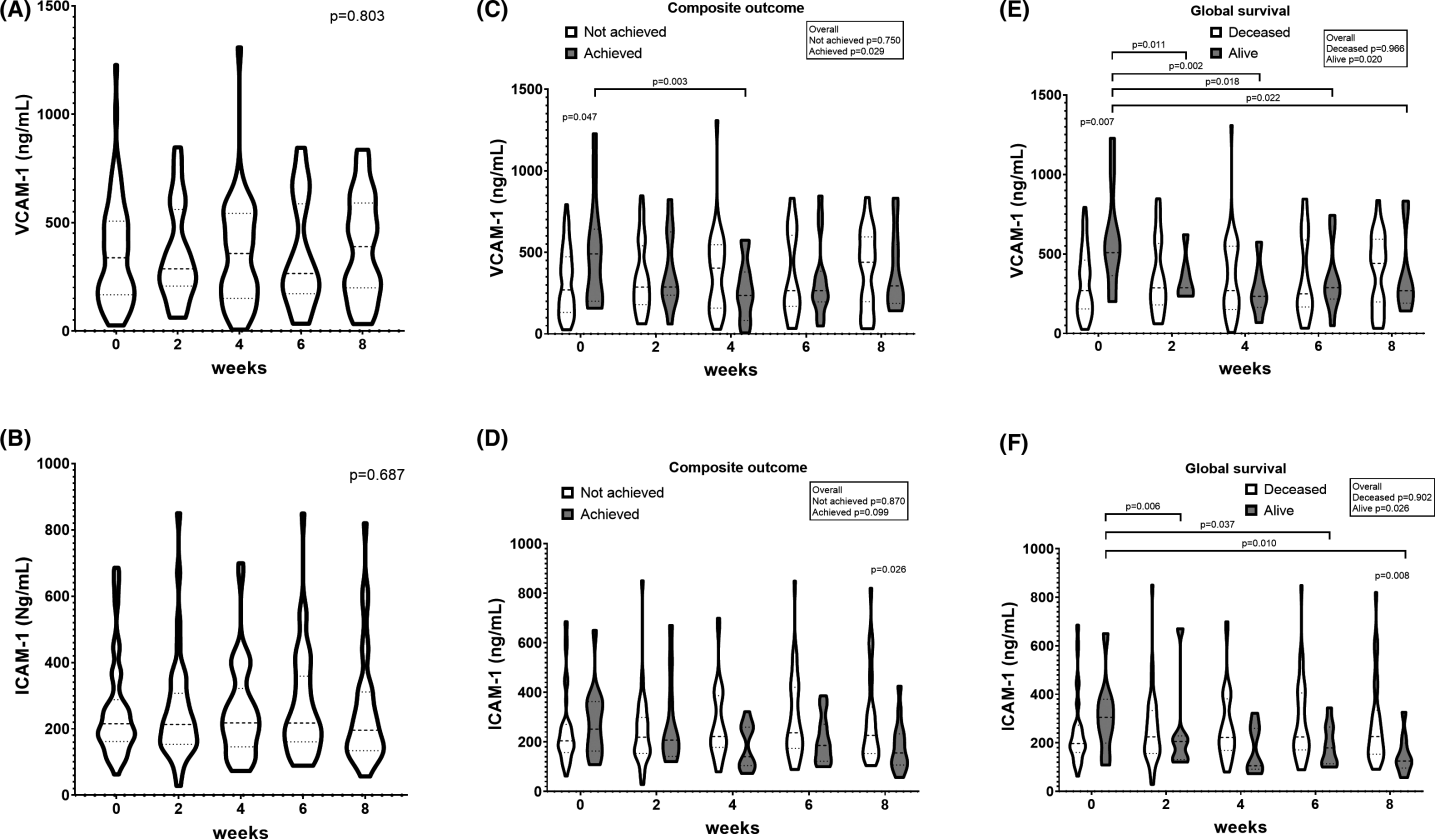
Serum levels of Cell Adhesion Molecules (CAMs) at baseline (time point 1) and across different cycles of nivolumab therapy (weeks 2 to 8). Vascular cellular adhesion molecule‐1 (VCAM‐1) serum levels in the overall cohort (A) and in the cohort divided according to the achievement of the composite outcome (C) and the global survival (E). Intracellular adhesion molecule‐1 (ICAM‐1) serum levels in the overall cohort (B) and in the cohort divided according to the achievement of the composite outcome (D), and the global survival (F). Data are expressed as median and interquartile range (at baseline *n* = 9 alive at the closing of the follow‐up; *n* = 14 achieved the composite outcome; *n* = 62 deceased during clinical follow‐up)

Considering the overall cohort, no significant variation was observed at different time points for both VCAM‐1 and ICAM‐1 (Figure 1A,B).

However, only in survivors, a significant reduction in both VCAM‐1 and ICAM‐1 serum levels was shown during treatment time points as compared to baseline (Figure 1E,F). After correction for repeated measures, the significance of changes in survivors was preserved for VCAM‐1 only (*p* = .025 for VCAM‐1 and *p* = .074 for ICAM‐1). Compared to the baseline level, a significant change in VCAM‐1 levels was detected for every time point, whereas the change for ICAM‐1 was significant for the fourth and the last time points only.

The composite outcome was achieved by 14 patients (19.7%); of them, 2 patients were followed for less than 2 years (528 and 471 days, respectively). A significant reduction of VCAM‐1 levels was observed in patients achieving the composite outcome between the baseline and the third time point, whereas no significant variation was detected for ICAM‐1 levels in both groups (Figure 1C,D).

### 3.3 Baseline values of VCAM‐1 and its change during treatment correlate with overall survival

Of 71 patients included in the analysis, 18 patients died and 10 patients discontinued the treatment on advice of their oncologist before the first radiological response evaluation. As a result, the complete profile of five measurements was available for 42 patients. The median follow‐up was 815.0 days (IQR: 651.5–990.5 days). The unadjusted OR for the composite outcome and the HR for OS, estimated for baseline levels of VCAM‐1, for its change between the baseline and the third time point and for potential confounders, are shown in Table 3. No potential confounder reached the sufficient statistical significance to enter a logistic regression model having the composite endpoint as dependent variable. The goodness‐of‐fit of logistic regression models have been confirmed through ROC curves (Figure S3). According to the ROC analysis, a reduction of VCAM‐1 144 ng/ml between the first and the third cycle of therapy predicts our composite outcome with a sensitivity 81.3% and a specificity of 81.8%.

**FIGURE 2:**
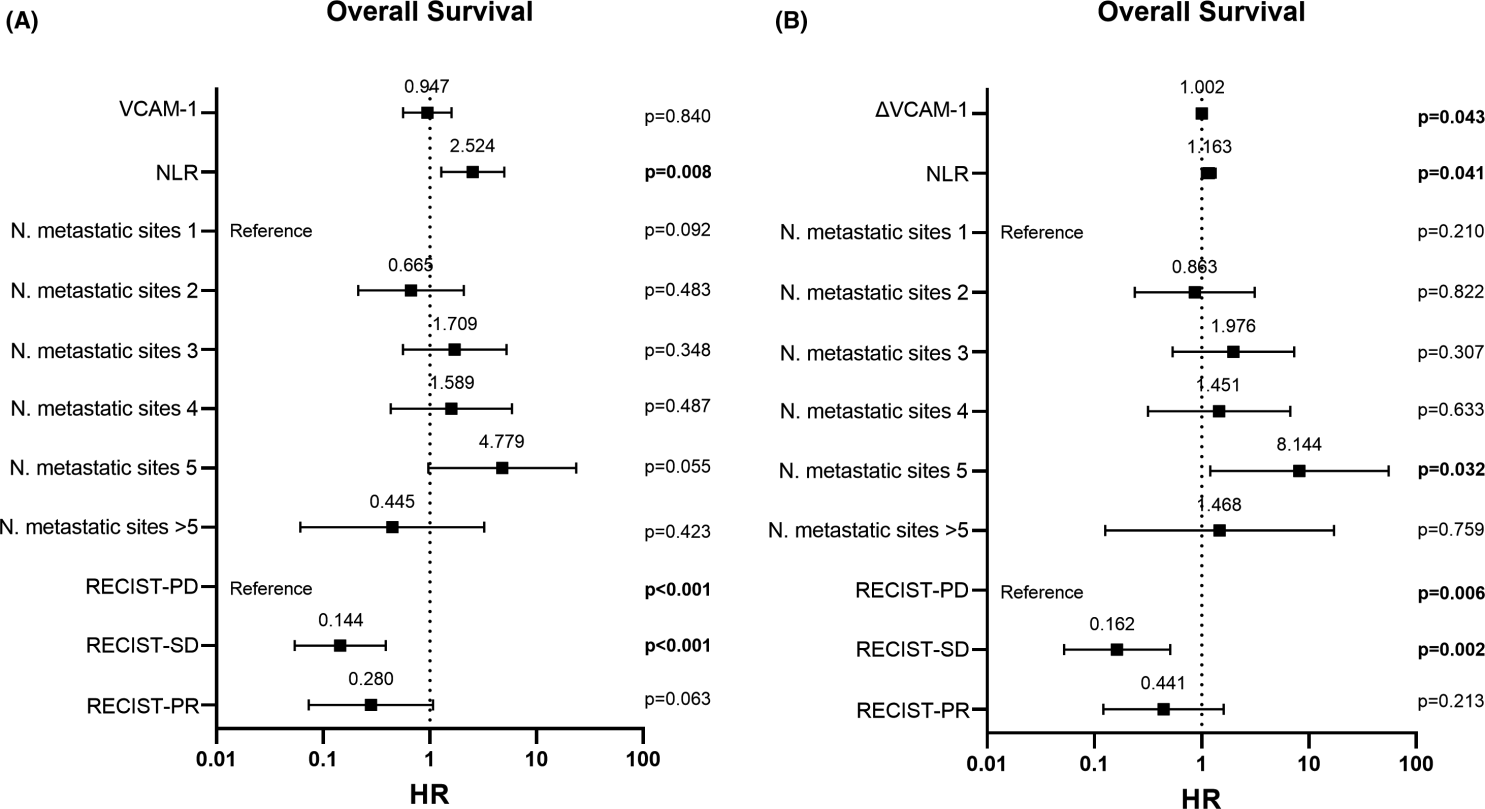
Multivariate Cox proportional regression models. The dependent variable is overall survival. Censoring event is as follows: death. Statistically significant p‐values are highlighted in bold. NLR, neutrophils‐to‐lymphocytes ratio; PD, progression of disease; PR, partial remission; RECIST, response evaluation criteria in solid tumors; SD, stable disease; VCAM‐1, vascular cellular adhesion molecule‐1

Performing multivariate Cox regression models, only the association between the change in VCAM‐1 over time and the OS was statistically significant after the adjustment for potential confounders (Figure 2).

## 4 DISCUSSION

The results of this study draw two main outlines about the potential role of VCAM‐1 in patients undergoing nivolumab treatment for NSCLC. As first, baseline levels of circulating VCAM‐1, as well as their variation during treatment, might be associated with the two‐year survival and the OS. The prognostic role of CAMs has been already proposed by some authors, according to clinical and pre‐clinical evidence. Basically, overexpression of CAMs is regarded as a negative prognostic factor, since it has been associated with a more aggressive phenotype^1^ and therapeutic failure of chemotherapy.^2^ Surprisingly, in our cohort, overexpression of VCAM‐1, shows a positive prognostic value. We may then speculate that circulating VCAM‐1, although being per se associated with a more aggressive disease, positively influences the response to nivolumab treatment, turning into a positive prognostic factor in this specific subset of patients. Such paradox could be explained by the dual biologic role of CAMs: on one side, they mediate neoplastic invasion and metastatic dissemination^11^; on the other hand, they promote extravasation of leukocytes, particularly pro‐inflammatory lymphocytes,^12^ which are targeted by nivolumab. Accordingly, higher levels of ICAM‐1 are associated with a higher expression of PD‐L1.

So, we can hypothesize that VCAM‐1 overexpression may enhance the immune‐mediated response to neoplastic tissues, promoted by nivolumab. An interaction between CAMs and PD‐L1 is biologically plausible, although the expression of PD‐L1 was not significantly associated with any of the endpoints. In this regard, we must acknowledge that the expression of PD‐L1 was available only for a small proportion of patients (33 patients, 46.5% of the overall population).

Regardless of pathophysiological mechanisms, this result has potential relevant practical drawbacks since biomarkers of response to nivolumab are warmly awaited by clinicians, in order to stratify the probability of treatment success. Indeed, available predictors (eg PD‐L1 expression and amplification, tumour mutation burden and infiltrating lymphocytes) may be burdened by poor prognostic performance, or high cost of the assay, and often require a biopsy to be determined.^13^ Conversely, blood and serum biomarkers are generally less expensive and are easily accessible, so they can be monitored during the treatment. CD4^+^ T cells, NLR and myeloid‐derived suppressor cells have been proposed as potential blood‐derived biomarkers.^14^, ^15^ Previous results of our group outlined the potential prognostic role of some inflammation‐related biomarkers, such as osteopontin, resistin and pro‐convertase subtilisin‐kexin 9 (PCSK9).^6^, ^16^, ^17^ Besides, interleukin 6 and cell‐free DNA have been proposed as prognostic serum biomarkers.^18^ The evidence about these serum biomarkers is still scarce, so a performance comparison among these potential biomarkers is not possible at the moment.

The second relevant outline is the observation of a reduction of CAMs expression in surviving patients, compared to non‐survivors. As no previous study has explored the effect of PD‐1 blockade on CAMs expression in NSCLC, no comparison with expected results can be performed. Likely, the observed reduction of CAMs expression is the result of a complex interaction between the malignancy and the immune system of the host. Further clinical and in vitro studies are expected to clarify these mechanisms.

In a future perspective, modification of VCAM‐1 expression could become a novel parameter for patient stratification and potentially even a new therapeutic target for maximizing the response to ICI in the treatment of NSCLC.

Despite these interesting insights, some study limitations should be acknowledged. Due to the small sample size, the result of the present study may be biased by the poor study power. In particular, the analysis of CAMs serum level variation during treatment could be affected by the high rate of early death and treatment discontinuation. As a consequence, this work should be considered as a preliminary study and any conclusion shall be confirmed by larger studies. This notwithstanding, both the primary and the secondary endpoint of the study were achieved. Secondly, the patients were not thoroughly screened for conditions that can modify the serum levels of CAMs, other than lung cancer, such as dyslipidemia and systemic atherosclerosis.^19^ Moreover, the enrolled population is mainly composed of patients with adenocarcinoma and these results could be not confirmed in patients with squamous NSCLC. Indeed, significant differences in the prognostic power of biomarkers have been described for squamous and non‐squamous NSCLC in regard of response to treatment with ICI.^20^ Furthermore, early response to treatment was assessed using the canonical RECIST criteria instead of revised criteria for immunotherapy, leading to a potential underestimation of favourable response to the treatment.^21^ Ultimately, the results of the study were obtained in patients receiving immunotherapy after first‐line chemotherapy, which is no longer the standard clinical practice; further studies are needed to investigate the prognostic value of CAMs in patients receiving immunotherapy as first‐line treatment, also taking into account the potential correlation with PD‐L1 expression, which was measured only in a small proportion of patients of the present study.

In conclusion, the results of this preliminary study show that high baseline serum levels of VCAM‐1 are associated with a longer survival in patients treated with nivolumab as second‐line treatment for NSCLC. Interestingly, surviving patients also experienced a significant reduction in VCAM‐1 serum levels during the treatment. Although the biological and clinical meaning of this phenomenon needs to be clarified, this could be used in clinical setting as an early prognostic marker.

## CONFLICT OF INTERESTS

Francesco Grossi has consulting/advisory relationship with and received honoraria from Bristol‐Myers Squibb, Merck Sharp & Dohme, and Pierre Fabre and has consulting/advisory relationship with AstraZeneca and Roche. Carlo Genova received honoraria from AstraZeneca, Boehringer‐Ingelheim, Bristol‐Myers‐Squibb. Erika Rijavec received honoraria from Bristol‐Myers Squibb. Paolo Spallarossa has consulting/advisory relationship with Incyte, Teva, Bristol‐Myers Squibb and received honoraria from Incyte, Teva and Servier. Marco Tagliamento declares travel, accommodations, expenses supported by Roche, Bristol‐Myers Squibb, Astra Zeneca, Takeda, and activity as medical writer supported by Novartis, Amgen outside the submitted work. Dr. Bonaventura received a travel grant from Kiniksa Pharmaceuticals Ltd. to attend the 2019 AHA Scientific Sessions and currently receives honoraria from Effetti s.r.l. (Milan, Italy) to collaborate on the medical website www.inflammology.org. The other authors indicated no financial relationships.

## Supporting information

Figure S1

Figure S2

Figure S3

Tables S1‐S3
