# Supplementary figures and images for: Serum levels of VCAM‐1 are associated with survival in patients treated with nivolumab for NSCLC
Source: Eur J Clin Invest. 2021 Aug 22;52(1):e13668. doi: 10.1111/eci.13668 (PMC9286788; doi:10.1111/eci.13668)

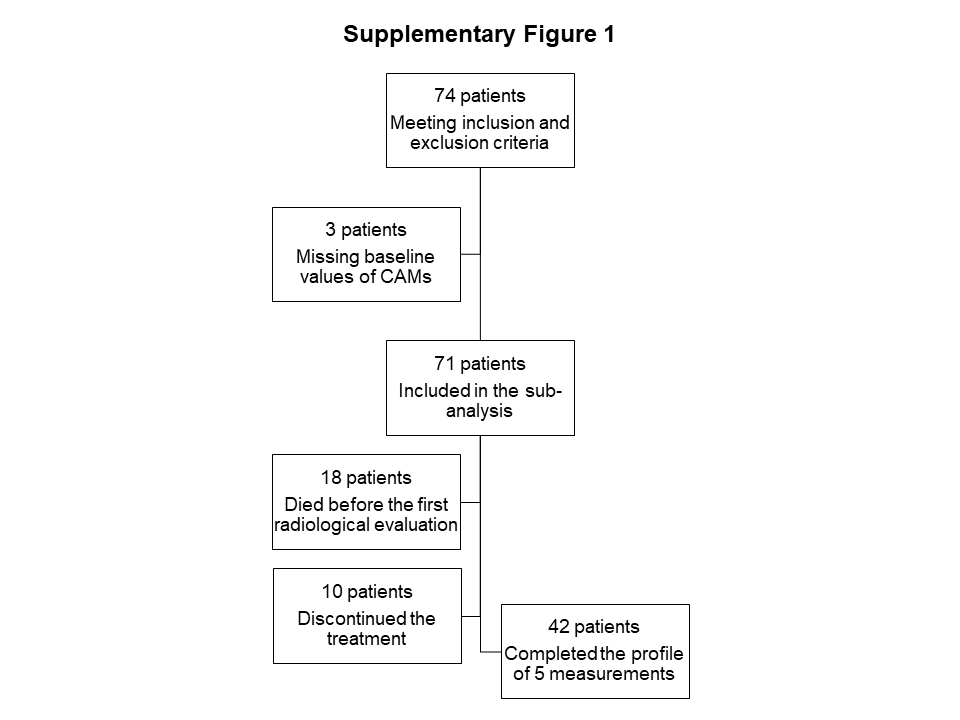

Supplement: Supplementary file 1 — Figure S1 [file ECI-52-0-s004.tif]

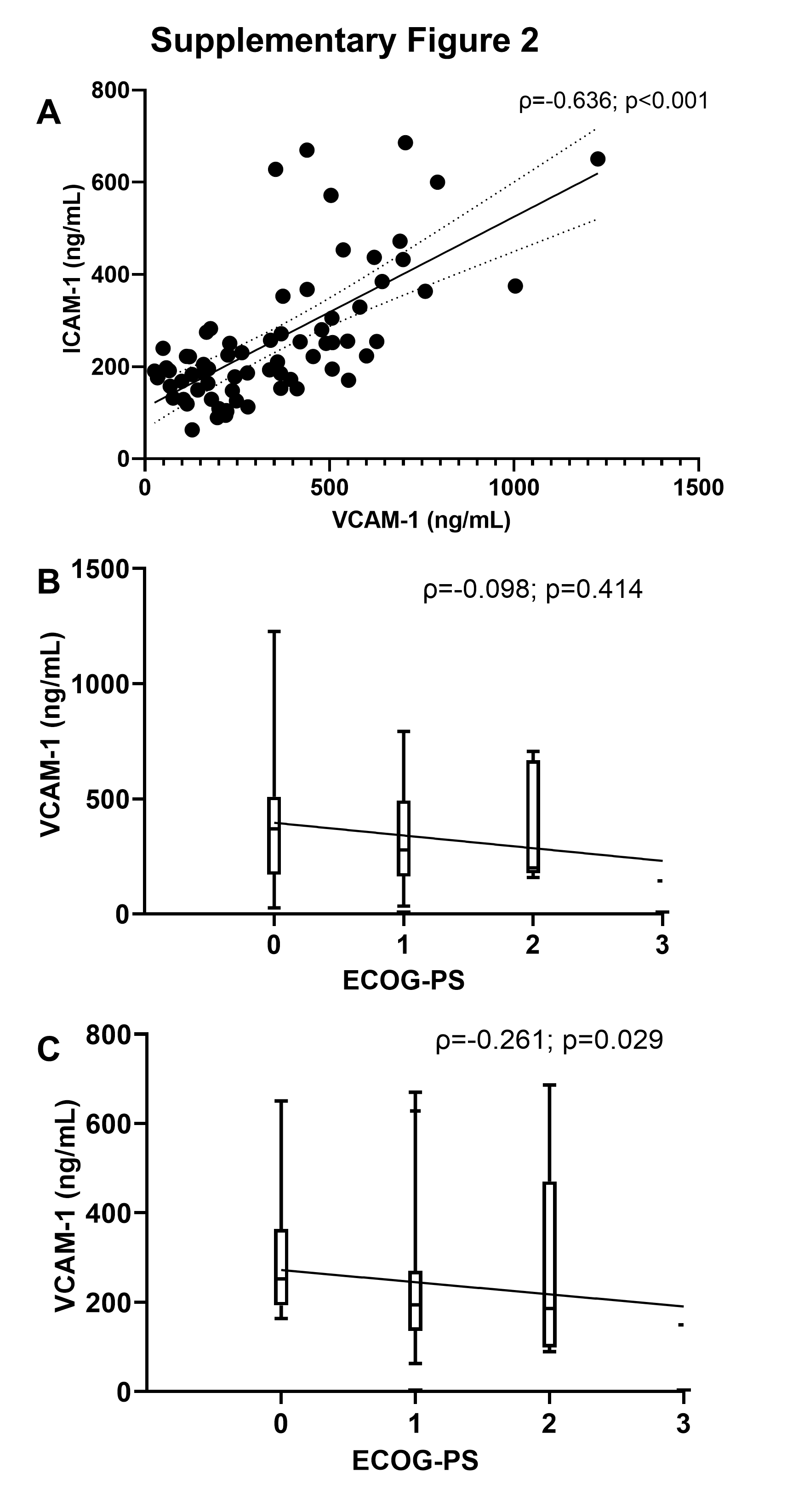

Supplement: Supplementary file 2 — Figure S2 [file ECI-52-0-s002.tif]

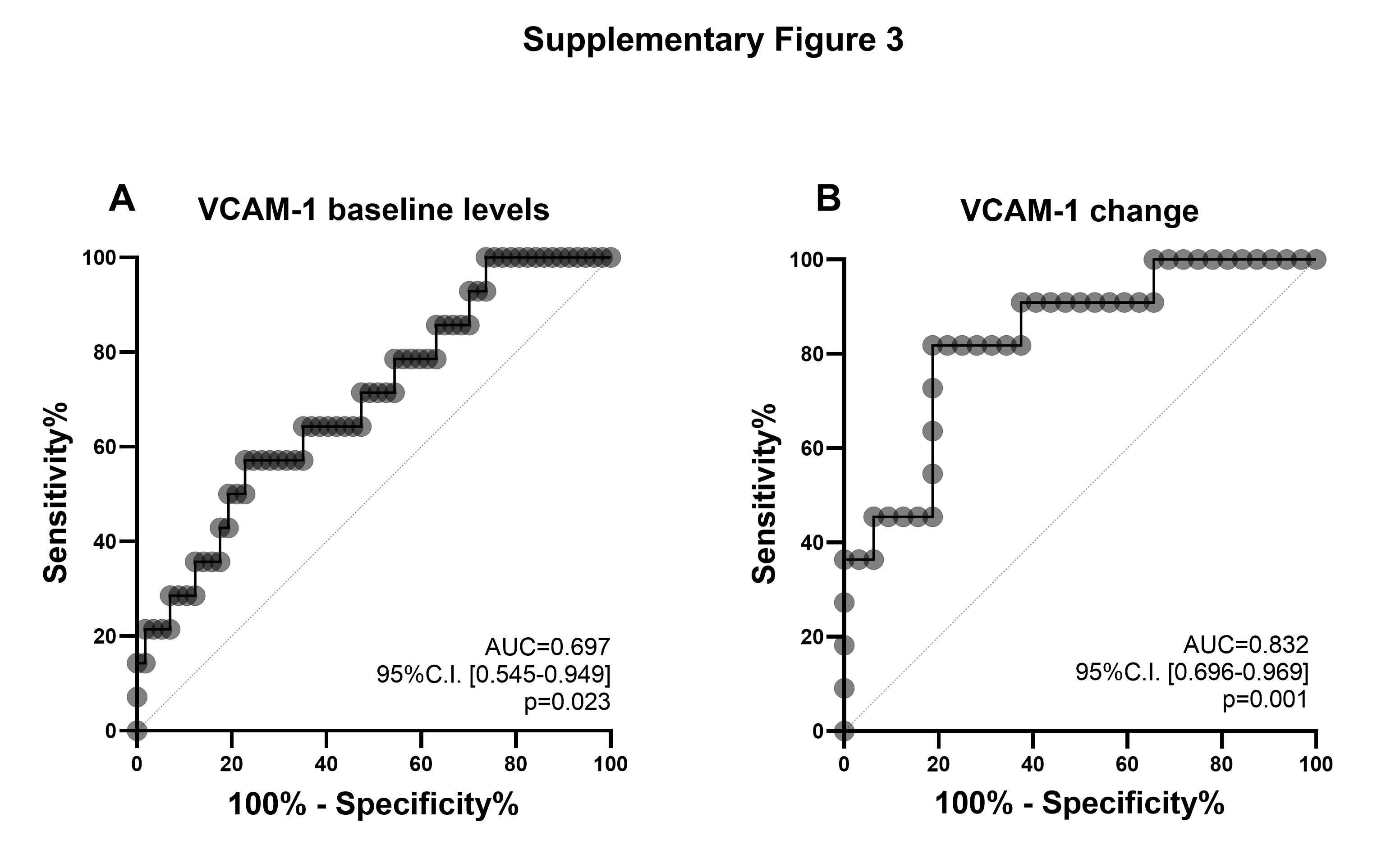

Supplement: Supplementary file 3 — Figure S3 [file ECI-52-0-s003.tif]
