# Supplementary material for: Serum levels of VCAM‐1 are associated with survival in patients treated with nivolumab for NSCLC
Source: Eur J Clin Invest. 2021 Aug 22;52(1):e13668. doi: 10.1111/eci.13668 (PMC9286788; doi:10.1111/eci.13668)
Supplement: Supplementary file 4 — Tables S1‐S3 [file ECI-52-0-s001.docx]

**Table S1. Descriptive statistic of clinical parameters at enrollment.**

|  | **Overall**  **(n=71)** |
| --- | --- |
| Age, years [IQR] | 70 (62-76) |
| Sex, male (%) | 50 (70.4) |
| Histology |  |
| ADC, n (%) | 52 (74.3) |
| SCC, n (%) | 16 (22.9) |
| Other, n (%) | 2 (2.9) |
| N. of metastatic sites |  |
| 1 | 8 (11.3) |
| 2 | 16 (22.5) |
| 3 | 21 (29.6) |
| 4 | 13 (18.3) |
| 5 | 6 (8.5) |
| >5 | 6 (8.5) |
| ECOG PS |  |
| 0, n (%) | 23 (32.4) |
| 1, n (%) | 42 (59.2) |
| 2, n (%) | 5 (7.0) |
| 3, n (%) | 1 (1.4) |
| Prior lines of treatment, n (%) |  |
| 1, n (%) | 32 (47.8) |
| 2, n (%) | 15 (22.4) |
| 3, n (%) | 12 (17.9) |
| 4, n (%) | 6 (9.0) |
| 5, n (%) | 0 (0.0) |
| 6, n (%) | 2 (3.0) |
| Smoking |  |
| Never, n (%) | 7 (10.8) |
| Former, n (%) | 24 (36.9) |
| Active, n (%) | 34 (52.3) |
| Pack/years, n [IQR] | 45 [32-79] |
| RECIST First response, n (%) |  |
| Early death, n (%) | 17 (25.4) |
| PD, n (%) | 28 (38.0) |
| SD, n (%) | 20 (28.2) |
| PR, n (%) | 6 (8.5) |
| CR, n (%) | 0 (0.0) |
| Death, n (%) | 62 (87.3) |
| OS, days (IQR) | 241 (63-514) |

Continuous variables are presented as median interquartile range [IQR], whereas categorical as absolute count (percentage).

ADC: adenocarcinoma; SCC: squamous cell carcinoma; ECOG PS: Eastern Cooperative Oncology Group Performance Status; RECIST: response evaluation criteria in solid tumors PD: progression disease; SD: stable disease; PR: partial response; CR: complete response; PFS: progression free survival; OS: overall survival.

**Table S2. Hematological and biochemical variables at enrollment.**

|  | **Overall**  **(n=71)** |
| --- | --- |
| Hb, g/dL [IQR] | 12.4 [10.7-13.2] |
| PLT, nx10^9^ [IQR] | 288 [223-335] |
| WBC, nx10^9^ [IQR] | 8.7 [7.0-11.7] |
| Neutrophils, nx10^9^ [IQR] | 6.5 [4.7-8.9] |
| Lymphocyte, nx10^9^ [IQR] | 1.2 [0.9-1.8] |
| Monocytes, nx10^9^ [IQR] | 0.6 [0.4-0.8] |
| Neutrophils-to-lymphocytes ratio, [IQR] | 5.4 [2.7-9.2] |
| Creatinine, mg/dL [IQR] | 0.9 [0.8-1.2] |
| CKD-EPI, mL/min [IQR] | 82 [61.92] |
| BUN, mg/dL [IQR] | 40 [32-48] |
| AST, U/L [IQR] | 19 [15-25] |
| ALT, U/L [IQR] | 16 [11-26] |
| ALP, U/L [IQR] | 87 [71-128] |
| γ-GT, U/L [IQR] | 46 [26-92] |
| LAD, U/L [IQR] | 228 [191-290] |
| Amylase, U/L [IQR] | 27 [16-41] |
| Lipase, U/L [IQR] | 27 [16-39] |
| Glucose, mg/dL [IQR] | 98 [89-125] |
| Albumin, g/L [IQR] | 38.5 [34.8-43.0] |
| CYFRA, ng/mL [IQR] | 7.0 [2.6-22.9] |
| NSE, ng/mL [IQR] | 8.0 [6.8-12.4] |
| ICAM-1, ng/mL [IQR] | 216 [162-288] |
| VCAM-1, ng/mL [IQR] | 338 [167-507] |

Hb: hemoglobin; PLT: platelet; WBC: white blood cell; BUN: blood urea nitrogen; ICAM: intercellular adhesion molecule; VCAM: vascular cell adhesion molecule.

**Table S3. ICAM-1/VCAM baseline correlation with other serum biomarkers.**

|  | **ICAM-1** | | **VCAM-1** | |
| --- | --- | --- | --- | --- |
|  | **ρ** | ***p*-value** | **ρ** | ***p*-value** |
| Hb | -0.106 | 0.382 | 0.182 | 0.128 |
| PLT | 0.129 | 0.286 | -0.001 | 0.993 |
| WBC | 0.047 | 0.701 | -0.071 | 0.554 |
| Neutrophils | 0.109 | 0.371 | -0.052 | 0.669 |
| Lymphocyte | -0.044 | 0.726 | -0.008 | 0.951 |
| Monocytes | -0.010 | 0.937 | -0.041 | 0.742 |
| Neutrophils-to-lymphocytes ratio | 0.087 | 0.485 | -0.084 | 0.501 |
| Creatinine | -0.029 | 0.812 | 0.145 | 0.227 |
| CKD-EPI | 0.008 | 0.947 | -0.119 | 0.323 |
| Uric acid | -0.040 | 0.739 | 0.135 | 0.262 |
| BUN | 0.073 | 0.549 | 0.186 | 0.121 |
| AST | 0.054 | 0.658 | -0.075 | 0.533 |
| ALT | 0.061 | 0.617 | -0.110 | 0.360 |
| ALP | 0.224 | 0.064 | 0.091 | 0.454 |
| γ-GT | 0.002 | 0.985 | -0.012 | 0.918 |
| LAD | 0.000 | 0.999 | -0.063 | 0.599 |
| Amylase | 0.131 | 0.309 | -0.036 | 0.780 |
| Lipase | 0.145 | 0.260 | 0.075 | 0.561 |
| Glucose | -0.107 | 0.381 | -0.008 | 0.948 |
| Albumin | -0.130 | 0.285 | 0.006 | 0.963 |
| CYFRA | -0.045 | 0.736 | -0.249 | 0.053 |
| NSE | 0.171 | 0.206 | 0.229 | 0.087 |

ICAM: intercellular adhesion molecule; VCAM: vascular cell adhesion molecule; Hb: hemoglobin; PLT: platelet; WBC: white blood cell; BUN: blood urea nitrogen;
